# Factors Affecting Length of Stay Following Elective Anterior and Posterior Cervical Spine Surgery

**DOI:** 10.7759/cureus.1452

**Published:** 2017-07-10

**Authors:** Frank J Yuk, Akbar Y Maniya, Jonathan J Rasouli, Alexa M Dessy, Patrick J McCormick, Tanvir F Choudhri

**Affiliations:** 1 Icahn School of Medicine at Mount Sinai, Mount Sinai Medical Center; 2 Anesthesiology, Memorial Sloan-Kettering Cancer Center

**Keywords:** length of stay, cervical spine surgery, anterior approach, posterior approach

## Abstract

Background

Disease of the cervical spine is widely prevalent, most commonly secondary to degenerative disc changes and spondylosis.

Objective

The goal of the paper was to identify a possible discrepancy regarding the length of stay (LOS) between the anterior and posterior approaches to elective cervical spine surgery and identify contributing factors.

Methods

A retrospective study was performed on 587 patients (341 anterior, 246 posterior) that underwent elective cervical spinal surgery between October 2001 and March 2014. Pre- and intraoperative data were analyzed. Statistical analysis was performed using GraphPad Prism 5 (GraphPad Software, Inc., La Jolla, CA) and the Statistical Package for Social Sciences (SPSS) (IBM SPSS Statistics, Armonk, NY).

Results

Average LOS was 3.21 ± 0.32 days for patients that benefited from the anterior approach cervical spinal surgery and 5.28 ± 0.37 days for patients that benefited from the posterior approach surgery, P-value < 0.0001. Anterior patients had lower American Society of Anesthesiologists scores (2.43 ± 0.036 vs. 2.70 ± 0.044). Anterior patients also had fewer intervertebral levels operated upon (2.18 ± 0.056 vs. 4.11 ± 0.13), shorter incisions (5.49 ± 0.093 cm vs. 9.25 ± 0.16 cm), lower estimated blood loss (EBL) (183.8 ± 9.0 cc vs. 340.0 ± 8.7 cc), and shorter procedure times (4.12 ± 0.09 hours vs. 4.47 ± 0.10 hours). Chi-squared tests for hypertension, coronary artery disease, congestive heart failure, chronic obstructive pulmonary disease, and asthma showed no significant difference between groups.

Conclusions:

Patients with anterior surgery performed experienced a length of stay that was 2.07 days shorter on average. Higher EBL, longer incisions, more intervertebral levels, and longer operating time were significantly associated with the posterior approach. Future studies should include multiple surgeons. The goal would be to create a model that could accurately predict the postoperative length of stay based on patient and operative factors.

## Introduction

The surgical treatment of degenerative disease of the cervical spine is one of the most commonly performed operations [1-2]. Surgical intervention is often required in cases where medical management has failed to alleviate symptoms [3]. Surgery can be accomplished through anterior, posterior, or combined approaches according to underlying pathology, each with variable postoperative hospital length of stay (LOS) [2]. Extended LOS is associated with an increased risk of complications, such as nosocomial infections [4], delirium [5], and venous thromboembolism [6]. A combination of preoperative and intraoperative patient-specific factors, such as age, opioid use, duration of surgery, and blood loss, have been correlated with prolonged hospital LOS. However, the question of how these factors can be influenced by the choice of surgical approach remains. Therefore, our study aims to connect these patient-specific factors to surgical approach and determine if hospital LOS can be influenced.

## Materials and methods

Patient selection

A total of 587 patients who underwent anterior (341) or posterior (246) cervical spine surgery between October 2001 and March 2014 were identified and their electronic medical records were retrospectively reviewed. All surgeries were performed at the Mount Sinai Medical Center by the senior author (TFC). The indications for surgery included disc herniation, degenerative disc disease, cervical spondylosis, tumor, cervical spondylotic myelopathy, cervical stenosis, and vertebral fractures. Excluded were patients undergoing emergent cervical spine surgeries immediately post-trauma and patients under 18 years old. Additionally, patients treated with combined or staged anterior and posterior surgeries were also excluded.

Data collection

Demographic data collected were age, gender, and body mass index (BMI) recorded at the time of surgery. Preoperative variables that were recorded included a history of major comorbidities, such as hypertension (HTN), coronary artery disease (CAD), valvular disease, congestive heart failure (CHF), chronic obstructive pulmonary disease (COPD), and asthma. Perioperative data collected from anesthetic records included the American Society of Anesthesiologists (ASA) score, operative time, and estimated blood loss (EBL). Operative time was determined as the period from the start of the procedure to the final closure of the skin. Intraoperative EBL was recorded as blood loss occurring during surgery, estimated from sponges, drapes, and suction containers. The number of intervertebral levels and incision length were recorded from the operative report. The LOS was determined from the time of surgery to the time of discharge to home or rehabilitation and recorded as an integer. Any patients discharged on the day of surgery were represented with a “0” day LOS. Therefore, LOS was defined by the number of calendar days, rather than every new 24-hour period.

Statistical analysis

Statistical analyses were conducted using GraphPad Prism 5 (La Jolla, CA, USA) and the Statistical Package for Social Sciences (SPSS) (IBM SPSS Statistics, Armonk, NY). The tests were two-tailed and statistical significance was determined using an α level of 0.05. Multiple linear regression was used to test the association between length of stay and demographic and intraoperative factors. To determine if the anterior and posterior patient populations were similar, unpaired t-tests (for continuous variables), the Mann-Whitney test (for ASA scores), and two-tailed χ2 or Fisher’s exact tests (for categorical variables) were used.

## Results

Comparison of patient and intraoperative factors

A total of 587 elective cervical spine surgeries (341 anterior, 246 posterior) were identified for analysis and met inclusion criteria. Of these patients, 293 were female and 294 were male. The average age of all patients was 55.08 ± 13.08 years (mean ± standard deviation) with a median of 55 years and range of 20-92 years. The average LOS was 4.08 ± 6.05 days (mean ± standard deviation) with a median of three days and range of 0 - 87 days.

**Figure 1:**
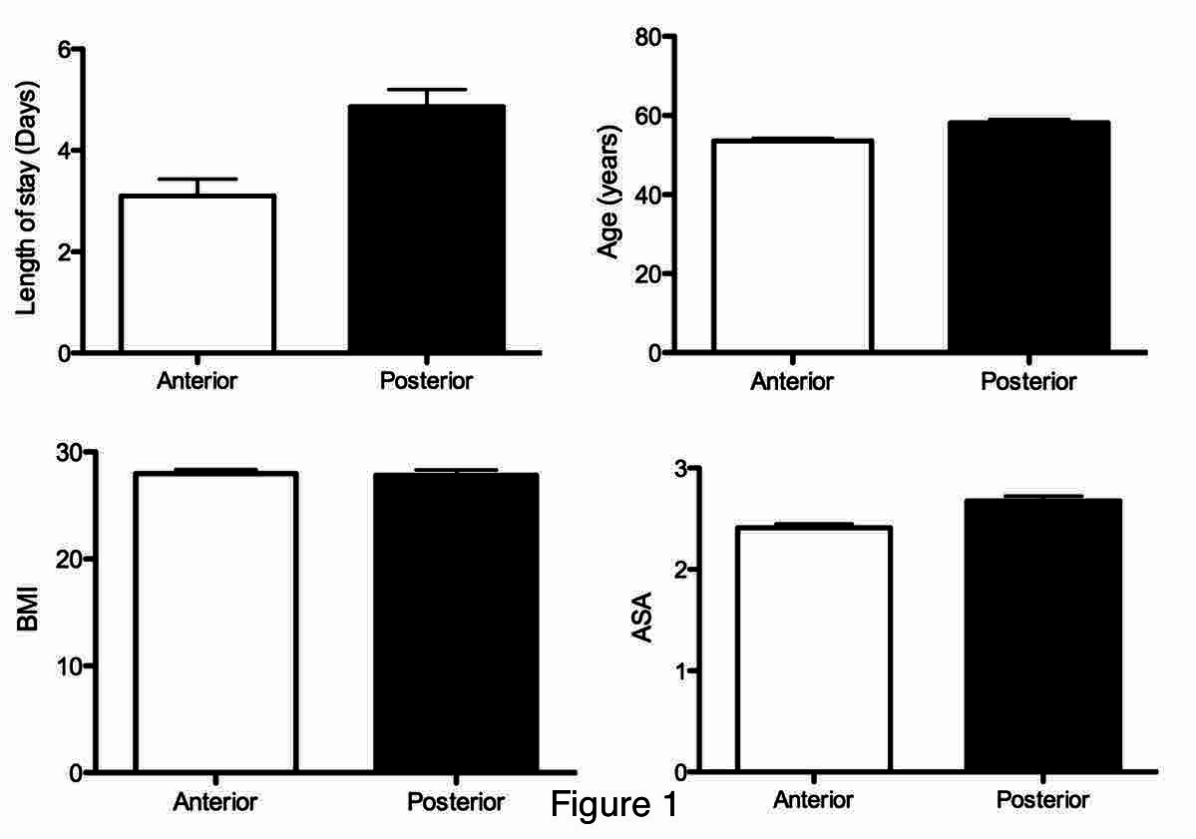
Comparison of length of stay (LOS), age, body mass index (BMI), and ASA scores in anterior versus posterior cervical spinal surgeries LOS: p < 0.002; age: p < 0.0001; BMI: not significant; ASA: p < 0.0001 ASA: American Society of Anesthesiologists

**Figure 2:**
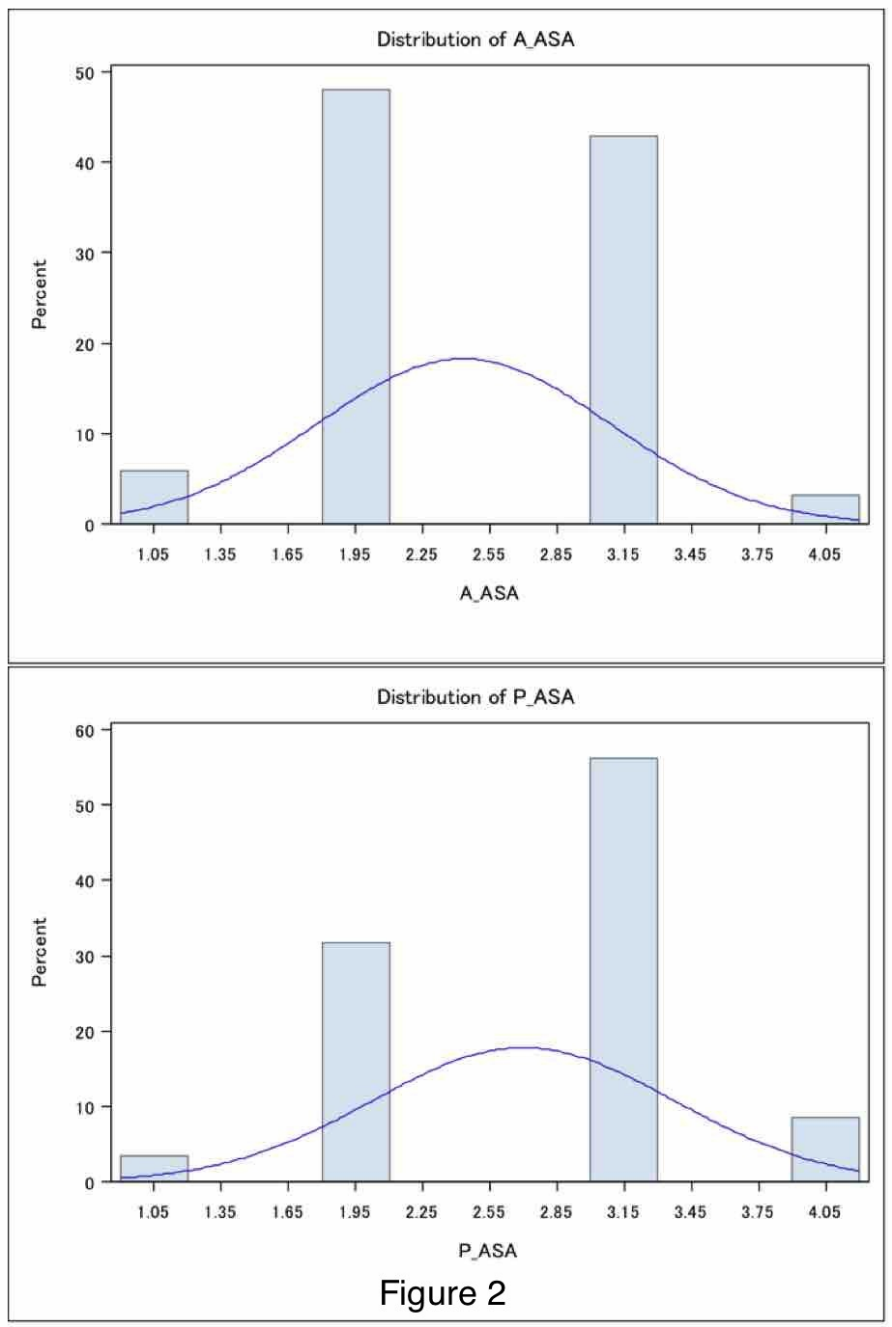
American Society of Anesthesiologists score distributions in anterior versus posterior cervical spinal surgeries Anterior (top): mean ± SE, 2.43 ± 0.036, median, 2; Posterior (bottom): mean ± SE, 2.70 ± 0.044, median, 3 SE: standard error

Patients receiving cervical spine surgery through an anterior approach had an average LOS of 3.21 ± 0.32 days (mean ± standard error (SE)) (Figure 1) with a range of 0-87 days and a median of two days and those receiving a cervical spine surgery through a posterior approach had a significantly longer average of 5.28 ± 0.37 days (mean ± SE; p < 0.0001, two-tailed t-test) with a range of 0 to 47 days and a median of four days. This was a 63.6% increase in the average. Further analysis revealed patients undergoing anterior cervical spine surgery were significantly younger (53.66 ± 0.69 years) when compared to 57.06 ± 0.85 years of posterior patients (mean ± SE, p = 0.0018) (Figure 1). However, a comparison of BMI showed no significant difference between patients (mean ± SE; kg/m2; anterior, 27.96 ± 0.34; posterior, 27.64 ± 0.39, p > 0.5) (Figure 1). Finally, ASA scores were significantly higher in the patients with posterior surgeries (mean ± SE; anterior, 2.43 ± 0.036; posterior, 2.70 ± 0.044; p < 0.0001, Mann-Whitney test (Figures 1-2), with median ASA scores of 2 and 3 for anterior and posterior approaches, respectively. Age was significantly associated with the ASA score in all patients (Pearson's r = 0.26; p < 0.0001). This held true when anterior approach patients were isolated (r = 0.31; p < 0.0001) and posterior approach patients were isolated (r = 0.18; p < 0.006).

**Figure 3:**
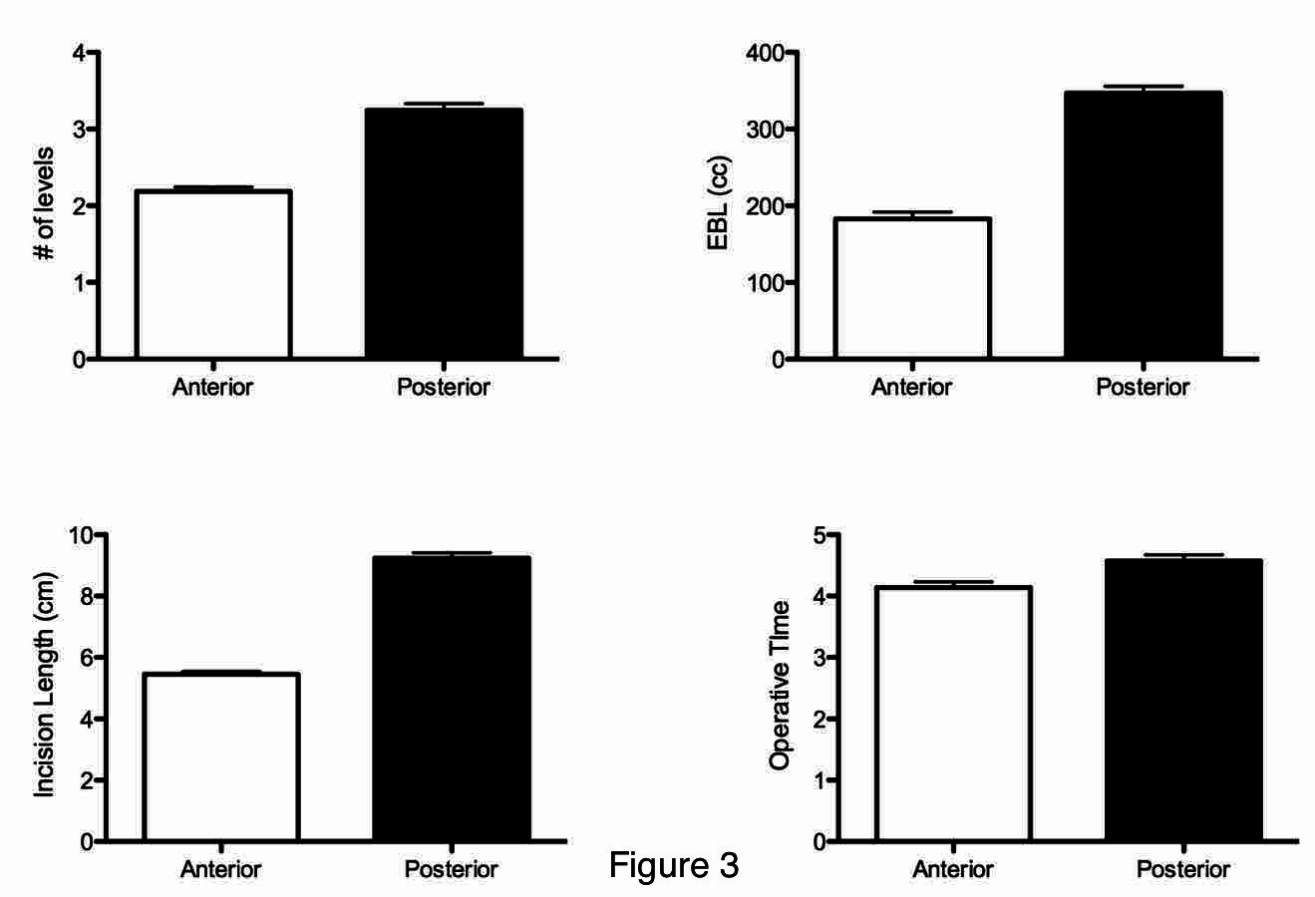
Comparison of number of levels, estimated blood loss (EBL), incision length, and operative time in anterior versus posterior cervical spinal surgeries Number of levels: p < 0.0001; EBL: p < 0.0001; incision length: p < 0.0001; operative time: p < 0.01

When comparing the number of intervertebral levels, patients receiving posterior spine surgeries had significantly more levels operated upon than patients receiving anterior spine surgeries (intervertebral levels; mean ± SE; anterior, 2.18 ± 0.056; posterior 4.11 ± 0.13; p < 0.0001) (Figure 3) with medians of two and four intervertebral levels for anterior and posterior approaches, respectively. Incision length was significantly longer in the posterior population (mean ± SE, median; anterior, 5.49 ± 0.093 cm, 6 cm; posterior, 9.25 ± 0.16 cm, 9 cm; p < 0.0001) (Figure 3) as well as EBL during surgery (mean ± SE; anterior, 183.3 ± 9.0 cc; posterior, 340.0 ± 8.7 cc; p < 0.0001) (Figure 3). Finally, surgery length was also significantly longer in the posterior approach than the anterior approach (mean ± SE; anterior, 4.12 ± 0.09 hours; posterior, 4.47 ± 0.10 hours; p < 0.01) (Figure 3). Linear regression model of all surgeries revealed a positive relationship between vertebral levels and incision length (r = 0.73; p < 0.0001) and incision length and vertebral level with surgery length (r = 0.40; p < 0.0001 and r = 0.41; p < 0.0001, respectively). Further, multivariable linear regression showed EBL was significantly associated with surgery length (r = 0.55; p < 0.0001), vertebral length (r = 0.44; p < 0.0001), and incision length (r = 0.60; p < 0.0001).

A review of patients’ medical histories and the subsequent Chi-squared test showed no difference in the number of patients with hypertension, coronary artery disease, valvular disease, congestive heart failure, COPD, and asthma between anterior and posterior patient populations.

Factors affecting anterior and posterior LOS

A multivariable linear regression of all patients, regardless of approach, revealed LOS was significantly associated with the number of vertebral levels (r = 0.17; p < 0.0001), incision length (r = 0.19; p < 0.0001), EBL (r = 0.27; p < 0.0001), ASA score (r = 0.22; p < 0.0001), and surgery length (r = 0.14; p < 0.002) (Table 1); however, no such association was found for age or BMI.

**Table 1:** Associations Between Length of Stay and Pre- and Intraoperative Factors Bold values denote statistical significance (p < 0.05). BMI: body mass index; ASA: American Society of Anesthesiologists; EBL: estimated blood loss.

	r – total	p-value	r – anterior	p-value	r – posterior	p-value
Age	0.06	0.11	0.14	< 0.02	-0.11	0.13
BMI	0.018	0.69	-0.031	0.59	0.11	0.16
ASA score	0.22	< 0.0001	0.25	< 0.0001	0.074	0.34
# of levels	0.17	< 0.0001	0.16	0.003	-0.046	0.56
Incision length	0.19	< 0.0001	0.22	0.001	0.0072	0.93
EBL	0.27	< 0.0001	0.13	0.01	0.24	0.002
Operative time	0.14	< 0.002	0.16	0.002	0.09	0.07

Further investigation of anterior surgeries showed LOS was significantly associated with age (r = 0.14; p < 0.02), ASA score (r = 0.25; p < 0.0001), number of intervertebral levels (r = 0.16; p < 0.004), incision length (r = 0.22; p = 0.0001), EBL (r = 0.13; p < 0.03), and surgery length (r = 0.16; p < 0.003). However, there was no significant association with BMI (Table 1). A similar analysis of posterior surgeries revealed LOS was significantly associated with EBL (r = 0.24; p = 0.0002) but not with age, BMI, ASA score, number of intervertebral levels, incision length, or surgery length.

## Discussion

In our study, patients who underwent anterior cervical spinal surgery stayed 2.07 days less on average than patients who underwent posterior surgeries. In general, this finding is consistent with the studies published to date [7-8]. Many patient specific factors could have contributed to this result, as there were multiple significant preoperative and intraoperative differences between the patient groups.

Investigators have identified numerous preoperative factors that increase the chances of a prolonged LOS, including advanced age [9-10], ASA score > 2 [9-11], higher BMI [10], gender, opioid use [12], intraoperative complications [13-15], and postoperative complications. These include cardiac, urinary, and pulmonary complications [13, 16-17]. Intraoperative factors adversely affecting LOS included extended operative time [18], blood transfusions [9, 19], adverse complications [20], and fibrin and drain use [21].

In our population, patients who underwent posterior surgery were older than those who underwent anterior surgery and had higher preoperative ASA scores. An increased ASA score was positively associated with increased LOS across all surgeries, and increased ASA score and age were positively associated with LOS for the anterior procedures. Increased age and ASA score have been identified in many studies as risk factors for increased LOS, postoperative complications, and morbidity and mortality [6, 10-14]. The results of this study substantiate those findings in the literature and suggest that the differences in age and ASA score could, in part, contribute to the increased LOS found in the posterior approach patients. Previous studies indicate that patients with a higher BMI generally have a longer LOS [10]. In our population of patients (with no significant difference in BMI between anterior and posterior groups), there was no association between BMI and LOS. This contrast with previous findings in the literature might result from differences in patient populations. Our study could also be inadequately powered to reveal such an association.

Patients in the posterior group had significantly more intervertebral levels operated upon, higher estimated blood loss, longer incisions, and longer procedures. These intraoperative factors were all positively associated with LOS across all patients and within the anterior group as well. These factors have all been associated with increased LOS in multiple studies in the spinal surgical literature [9, 18-19] and are, therefore, all possible contributors to the increased LOS found within this patient population.

The anterior and posterior groups contained no significant differences with regards to HTN, CAD, valvular disease, CHF, COPD, or asthma. Previous studies have identified these conditions as risk factors for increased LOS [13, 16-17]. Our results imply that either these factors independently increase LOS, even when these comorbidities are controlled for, or that the study was not adequately powered to reveal the differences in the comorbidities.

The primary limitations of this study are that it is retrospective and the data is from one surgeon at one institution. These make it difficult to be certain which pre- and intraoperative factors resulted in increased LOS in the posterior group of patients and limit the generalizability of the findings. Furthermore, separated analysis by indication for surgery (cervical spondylotic myelopathy, tumor, etc.) was not performed. There may be differences in LOS between these disease processes that were overlooked by this study.

## Conclusions

Patients with anterior surgeries performed stayed 2.07 days less on average postoperatively than patients with posterior surgeries performed. Higher EBL, longer incisions, more intervertebral levels, and longer operating times were significantly associated with the posterior approach. No differences between comorbidities were found between the two cohorts, but a subgroup analysis of LOS in patients with these diseases within the population will need to be performed in a future study.

A long-term goal would be to build a model to predict LOS based on factors specific to the patient as well as the procedure being performed. This will require the inclusion of multiple surgeons and institutions, along with an analysis of procedure type and the disease processes requiring treatment. Other factors that have been associated with LOS should be explored in future studies as well, postoperative pain being an example. Such a model would be invaluable to practitioners and hospitals alike.
